# Pericytes as targets in hereditary hemorrhagic telangiectasia

**DOI:** 10.3389/fgene.2015.00037

**Published:** 2015-02-13

**Authors:** Jérémy Thalgott, Damien Dos-Santos-Luis, Franck Lebrin

**Affiliations:** INSERM, Center for Interdisciplinary Research in Biology, UMR CNRS 7241/INSERM U1050, Group Pathological Angiogenesis and Vessel Normalization, Collège de FranceParis, France

**Keywords:** rare vascular disease, pericyte, thalidomide, angiogenesis, transforming growth factor-β signaling

## Abstract

Defective paracrine Transforming Growth Factor-β (TGF-β) signaling between endothelial cells and the neighboring mural cells have been thought to lead to the development of vascular lesions that are characteristic of Hereditary Hemorrhagic Telangiectasia (HHT). This review highlights recent progress in our understanding of TGF-β signaling in mural cell recruitment and vessel stabilization and how perturbed TGF-β signaling might contribute to defective endothelial-mural cell interaction affecting vessel functionalities. Our recent findings have provided exciting insights into the role of thalidomide, a drug that reduces both the frequency and the duration of epistaxis in individuals with HHT by targeting mural cells. These advances provide opportunities for the development of new therapies for vascular malformations.

## Introduction

Hereditary Hemorrhagic Telangiectasia (HHT) also known as Osler-Weber-Rendu syndrome is an autosomal dominant vascular disorder that affects 1 in 5000 individuals worldwide. The majority of HHT individuals will have HHT1 due to mutations in *ENG* encoding endoglin (McAllister et al., 1994) or HHT2 due to mutations in *ACVRL1* encoding Activin receptor Like-Kinase 1 (ALK1) (Johnson et al., 1996). Both are receptors for Transforming Growth Factor-β (TGF-β)/Bone Morphogenetic Protein (BMP) expressed primarily in endothelial cells. There are at least two further unidentified genes that can cause HHT, HHT3 on chromosome 5q (Cole et al., 2005; Govani and Shovlin, 2010) and HHT4 on chromosome 7p (Bayrak-Toydemir et al., 2006a). Finally, some *SMAD4* mutations can cause a syndrome comprising both juvenile polyposis and HHT phenotypes (Gallione et al., 2004) while *BMP9* mutations have been linked to vascular malformations that have phenotypic overlap with HHT (Wooderchak-Donahue et al., 2013). It is currently believed that in most if not all cases, HHT mutations represent null alleles, implying that the remaining wild-type allele is unable to contribute sufficient protein for normal vascular functions. Thus, the predominant mechanism underlying HHT phenotypes seems to be haploinsufficiency (Abdalla and Letarte, 2006).

Clinically, HHT is characterized by large arteriovenous malformations (AVMs) that are found in major organs including the lung, liver and brain. They consist of direct connections between arteries and veins without an intervening capillary bed. They can cause severe morbidity and mortality if not recognized and treated. Multiple red spots known as telangiectases are typically found in the nasal septum, oral mucosa and gastrointestinal tract. They consist of clusters of abnormally dilated thin-walled vessels that are prone to bleed with slight trauma. All classical features of HHT can be seen in both HHT1 and HHT2, but the prevalence of specific vascular malformations varies according to the genotype. Pulmonary and cerebral AVMs are more common in HHT1 than HHT2, 85 vs. 35% (van Gent et al., 2010) and 20 vs. 2% (Letteboer et al., 2006), respectively. HHT2 individuals have a higher incidence of hepatic AVMs (Bayrak-Toydemir et al., 2006a,b; Bossler et al., 2006; Lesca et al., 2007). The major quality of life issue for many individuals with HHT is frequent and severe nose and gastrointestinal bleeding from mucosal telangiectases that can cause severe anemia (Shovlin, 2010). Multiple lesions disseminated over the entire mucosal surface are common in affected individuals, making local treatment difficult. Therapeutic manipulation of coagulation and fibrinolytic pathways is often employed to try to limit blood loss in HHT. Recent randomized controlled trials have demonstrated the efficacy of tranexamic acid in the treatment of severe bleeds in individuals with HHT (Gaillard et al., 2014; Geisthoff et al., 2014). Aminocaproic acid may also be effective (Saba et al., 1994). Hormonal manipulation in the form of estrogen-progesterone regimen and tamoxifen has been shown to be beneficial in treating epistaxis (Van Cutsem et al., 1988, 1990, Yaniv et al., 2009). Surgical replacement of nasal epithelium by skin, argon laser coagulation or antioxidants is also used and shows efficacy (Sadick et al., 2003; Lesnik et al., 2007; de Gussem et al., 2009). However, all these options just offer a hemorrhage-free interval and have side effects (Shovlin, 2010) and alternatives are still a significant unmet need.

Accumulating data indicate that excessive angiogenesis is implicated in the pathogenesis of HHT and may contribute to the formation of AVMs (Xu et al., 2004; Park et al., 2009; Lebrin et al., 2010; Choi et al., 2012; Mahmoud et al., 2010; Choi et al., 2013; Chen et al., 2013), suggesting that angiogenesis inhibitors might be promising agents to treat HHT symptoms (Lebrin et al., 2010; Dupuis-Girod et al., 2012, 2014; Walker et al., 2012; Han et al., 2014; Riss et al., 2014). Angiogenesis involves the growth of new blood vessels from pre-existing ones (Carmeliet and Jain, 2011; Geudens and Gerhardt, 2011; Potente et al., 2011). The formation of new sprouts is highly dynamic and requires a multitude of highly orchestrated processes initiated by the selection of a fraction of endothelial cells that acquire a highly motile phenotype that become called endothelial Tip cells (Lobov et al., 2007; Jakobsson et al., 2010; Benedito et al., 2012). The other endothelial cells termed Stalk cells stay behind the Tip cell, proliferate and form the new tube to maintain the integrity and perfusion of the growing vascular bed (Eilken and Adams, 2010; Wacker and Gerhardt, 2011; Ribatti and Crivellato, 2012). The endothelial cell specification is highly controlled by a fined-tuned feedback loop between VEGF signaling and Notch/Dll4 signaling ensuring a “salt and pepper” distribution of endothelial Tip and Stalk cells within the activated endothelium (Ruhrberg et al., 2002; Gerhardt et al., 2003; Covassin et al., 2006; Hellström et al., 2007; Suchting et al., 2007; Tammela et al., 2008; Eilken and Adams, 2010; Jakobsson et al., 2010; Wacker and Gerhardt, 2011; Ribatti and Crivellato, 2012). New sprouts then extend, form a lumen and eventually meet and connect in a process called anastomosis creating a primitive vascular network that is further remodeled by regression and stabilization to support blood flow, become specialized as arteries and veins and recruit mural cells. One important factor regulating vessel remodeling is oxygen as elevated oxygen induces vessel pruning, ensuring that the vascular density is correctly adapted to the tissue demand. The recruitment of the mural cells, pericytes and vascular smooth muscle cells (VSMCs) that coat small capillaries and larger vessels respectively, marks the end of the plasticity time-window in vascular development during which pruning can occur. The prominent signaling pathways that regulate endothelial-mural cell-cell communication are Platelet Derived Growth Factor-β (PDGF-β)/PDGF Receptor-β, angiopoietin 1 (Ang1)/Tie2 and TGF-β, which control mural cell recruitment, endothelial cell viability and mural cell differentiation, respectively (Gaengel et al., 2009; Armulik et al., 2011; Stapor et al., 2014). Several possible mechanisms have been proposed to explain how mutations in *Acvrl1* or *Eng* gene may lead to aberrant angiogenesis. These include increased VEGF production (Cirulli et al., 2003; Sadick et al., 2005a,b, 2008), and inappropriate responses of mutated endothelial cells to TGF-β (Lebrin et al., 2004; Xu et al., 2004; Fernandez-L et al., 2005) or to BMP9/10 stimulation (Ricard et al., 2010, 2012; Kim et al., 2012; Young et al., 2012) that cause excessive endothelial cell proliferation and migration inhibiting vessel maturation. ALK1-Smad1/5 signaling cascade has also recently been reported to synergize with activated Notch in Stalk cells to repress Tip cell formation and endothelial sprouting thus establishing a robust Tip-Stalk cell selection (Larrivée et al., 2012; Moya et al., 2012). Consequently, blockage of ALK1 or BMP9 showed a denser more highly branched vascular plexus in retinas of post-natal P7 mice (Larrivée et al., 2012; Ricard et al., 2012). In agreement, mutant embryos lacking Smad1/5 specifically in the endothelium had excessive number of sprouts in the dorsal aorta at embryonic day E9.5 and died due to severe defective angiogenesis and lymphangiogenesis at E14.5 (Moya et al., 2012). Finally, another important consequence of impaired TGF-β/BMP signaling in endothelial cells might be defective endothelial-mural cell-cell communication due to reduced activation of TGF-β (Carvalho et al., 2004). It affects mural cell recruitment and vessel stabilization that leads to fragile blood capillaries. As consequence, they become prone to respond to angiogenic stimuli and to bleed with slight trauma, the pathological hallmark of HHT (Carvalho et al., 2004; Lebrin et al., 2010). The mechanisms underlying TGF-β/BMP mediated vessel maturation are not fully understood, although it has recently been demonstrated that thalidomide reduces the frequency and duration of nosebleeds in individuals with HHT by stimulating vessel maturation. This provides the first demonstration that strategies targeting mural cells of blood capillaries named pericytes can have beneficial effects on bleeding from vascular malformations (Lebrin et al., 2010). In this present review, we focus on recent insights into the mechanisms that regulate TGF-β/BMP mediated endothelial cell-pericyte communication, in particular how pericyte deficiencies may contribute to the pathogenesis of HHT. Finally, we will discuss the mechanisms of action of thalidomide and its potential for treating vascular malformations in HHT.

## Pericytes are obligatory constituents of blood capillaries

**Figure 1:**
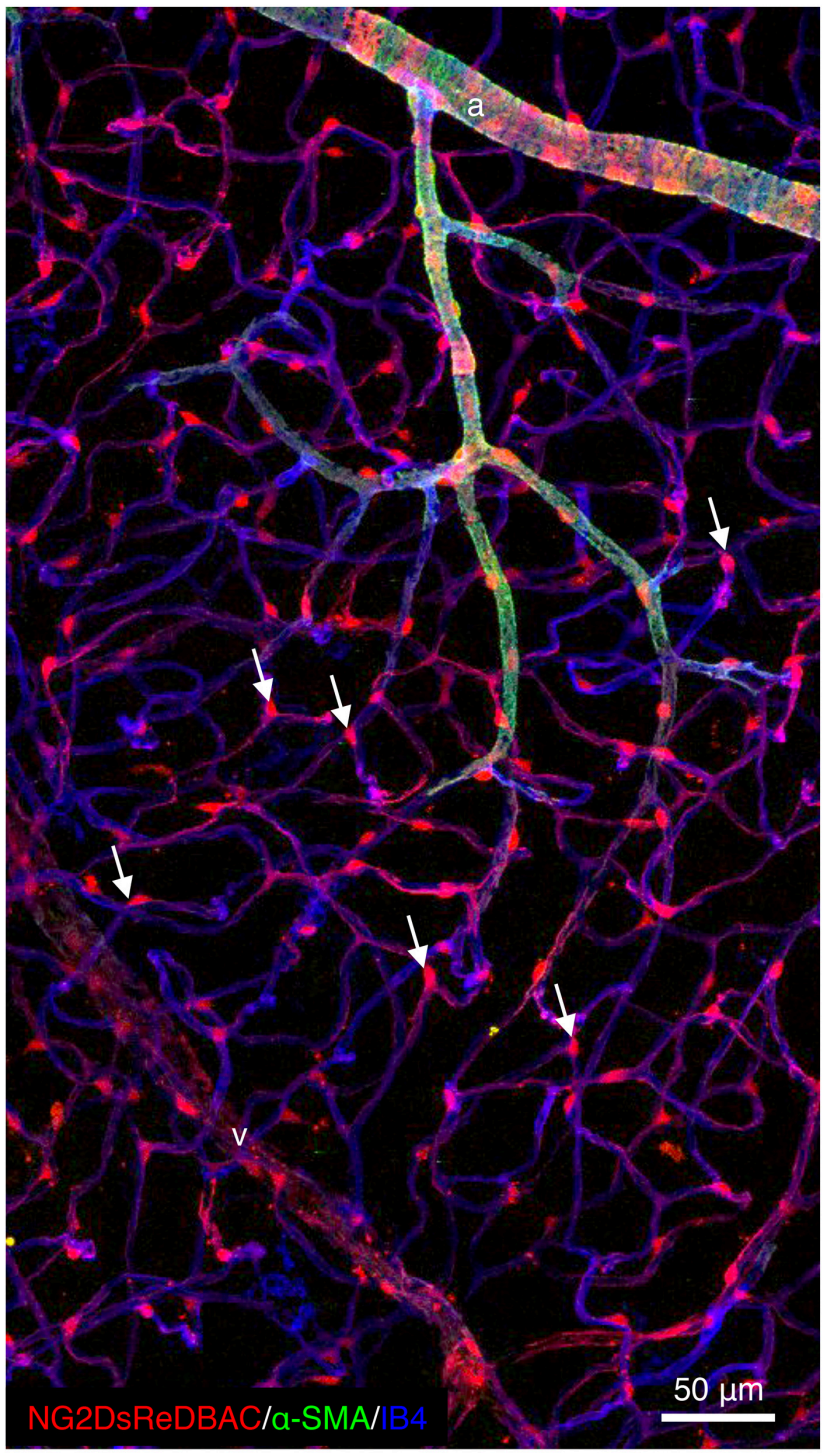
**Pericytes are ubiquitously present in blood capillaries**. Confocal images of retinas from adult NG2DsREDBAC-transgenic mice stained with isloectin-B4 and α-SMA to reveal the vascular plexus (in blue) and to label the VSMCs (in green), respectively. VSMCs cover the arterioles and have a flattened, spindle-shaped appearance with few cytoplasmic processes. Pericytes (in red) appear to be ubiquitously present in blood capillaries and extend primary processes along the abluminal surface of the endothelial tube. White arrows indicate pericytes that are found at the capillary branch points where they extend processes along each vessel branch conferring a cellular Y-shape.

Recent use of a combination of unique transgenic mice expressing fluorescent pericytes and high-resolution confocal imaging has permitted appreciation of the extent of pericyte heterogeneity throughout the microvasculature of all organs. In contrast to VSMCs that surround arteries and veins in multiple concentric layers of cells that are perpendicular to the direction of the blood flow and are separated from the vascular basal membrane (BM) by a layer of mesenchymal cells and extracellular matrix, pericytes are flattened, solitary and extend primary cytoplasmic processes along the abluminal surface of intermediate size to small vessels, contacting several endothelial cells. In particular, somas are often found at capillary branch points where pericytes extend primary processes along each vessel branch conferring a cellular Y-shape (Figure 1). Moreover, multiple cytoplasmic processes that extend perpendicularly from the primary processes are also detected and encircle the blood capillary increasing the area of contact with the abluminal surface of the endothelium. Importantly, the pericyte density and the endothelial abluminal surface covered by pericytes vary between different organs and different vascular beds. These correlate positively with vessel barrier properties. In agreement, the central nervous system (CNS) is the most covered tissue (Sims, 1986; Mathiisen et al., 2010; Armulik et al., 2011).

Pericytes are embedded within the vascular BM of terminal arterioles, capillaries and post-capillary venules, although they might be found in large vessels as well (Díaz-Flores et al., 2009). It is therefore important to remember that the distinction between pericyte and VSMC morphology and location is not absolute. A continuum of phenotypes ranging from classical VSMC to the typical pericyte distributed along the vessels reflects more the reality. Pericytes belong to the same lineage and category of cells than VSMC and are believed to differentiate into VSMC and *vice versa* in conjunction with vessel growth and remodeling (Nehls and Drenckhahn, 1993). Several markers have been used to identify pericytes and include Neural/glial antigen 2 (NG2), PDGFR-β, α-Smooth Muscle Actin (α-SMA), desmin, vimentin, aminopeptidase A or N and Regulator of G protein 5 (RGS5) (Armulik et al., 2005, 2011; Díaz-Flores et al., 2009; Krueger and Bechmann, 2010). Although no single exclusive pericyte-marker is known and all markers currently used are not specific and dynamic in their expression. They may be up or down regulated in conjunction with developmental states or pathological situations (Stapor et al., 2014). Indeed, α-SMA expression is restricted to pre and post capillary regions and is often absent in quiescent pericytes in normal tissues. However, its expression strongly increases in pericytes in pathological situations such as tumor angiogenesis, tissue fibrosis and inflammation (Gerhardt and Betsholtz, 2003). Interestingly, it has recently been suggested that a subpopulation of pericytes expressing specific markers such as high levels of NG2 and class III-β tubulin performs specific functions during sprouting angiogenesis (Stapor et al., 2014). However, the mapping of specific functions and downstream mechanisms to specific pericyte dynamic remains elusive. Does a subtype of pericytes implicated in sprouting angiogenesis exist *in vivo*? Or do pericytes change their morphology like endothelial Tip and Stalk cells in order to perform specific functions? Further studies are awaited to clarify these questions.

It is not surprising that for many years, studies of blood vessels have concentrated mainly on the endothelial component, especially with the discovery of VEGF as potential target in eye and cancer diseases (Potente et al., 2011). By comparison research focusing on the perivascular compartment has been relatively neglected, mainly because of the lack of specific markers. However, pericytes have recently gained increasing attention as obligatory constituents of blood microvessels and important regulators of vascular morphogenesis during development, vascular homeostasis and disease. They maintain the stability of the vasculature, regulate endothelial cell proliferation and survival (Gerhardt and Betsholtz, 2003; Gaengel et al., 2009; Armulik et al., 2011) and control capillary diameter and local blood flow (Peppiatt et al., 2006; Fernández-Klett et al., 2010; Hamilton et al., 2010; Hall et al., 2014). Emerging concepts also include the physiological role of pericytes in the regulation of vascular permeability to solutes, molecules and immune cells (Armulik et al., 2010; Daneman et al., 2010; Stark et al., 2013). Pericytes are implicated in the development of diabetic retinopathy (Beltramo and Porta, 2013), Alzheimer's disease (Sagare et al., 2013; Winkler et al., 2014) and is an obligatory component of the tumor stroma (Gaengel et al., 2009; Armulik et al., 2011). More recently, *PDGFB* and *PDGFRB* mutations have been linked to the development of an autosomal dominant rare disorder named Idiopathic Basal Ganglia Calcification (IBCG) (Keller et al., 2013; Nicolas et al., 2013). IBCG individuals display motor, cognitive and psychiatric symptoms. The mechanisms of the disease are not fully characterized, but the occurrence of the calcium deposition may correlate with the degree of pericyte and blood barrier deficiencies as they show in mice (Keller et al., 2013).

Due to their roles in health and diseases and their special characteristics, in particular those related to cell plasticity, pericytes might be potential drug targets for future therapies. However, we still lack understanding about many aspects of pericyte-endothelial cell communication and how the density, morphology and maturation stages of pericytes affect vessel functions. It is currently accepted that mature pericytes are cells embedded within the vascular BM that can make direct interactions with endothelial cells through specific contacts. The number and size of pericyte-endothelial cell contacts vary between tissues but up to 1000 contacts have been reported for one endothelial cell. They include *peg-pocket* types in which pericyte cytoplasmic fingers are inserted into endothelial invaginations, *occluding contacts* where two membranes come very close together and *adhesion plaques* that contain fibronectin and may be the sites where N-cadherin-based connections are formed (Gerhardt et al., 2000; Gerhardt and Betsholtz, 2003) and finally, *gap-junction-like structures* (Díaz-Flores et al., 2009; Li et al., 2011; Winkler et al., 2011). Together, these intimate interactions between pericytes and endothelial cells leave us with the notion that pericytes are distributed along the vasculature to facilitate, integrate and coordinate vessel functions. Interactions will involve paracrine and contact-dependent signaling. More generally, impairments of one vessel wall cell type will inevitably affect the other. In the next section, we will point out advances in the understanding of how TGF-β/BMP signaling pathways regulate vessel stability and in particular, how ALK1 or endoglin haploinsufficiency leads to defective pericyte-endothelial cell interactions.

## Defective TGF-β/BMP signaling in endothelial cells affects vessel stability and pericyte attachment

Classically, TGF-β and BMP signaling pathways are each initiated by ligand-mediated activation of distinct type I and type II serine/threonine kinase transmembrane receptors. Within the ligand-induced heteromeric receptor complex, the constitutively active type II receptor phosphorylates the type I receptor on specific serine/threonine residues in the intracellular juxtamembrane region named GS-domain leading to the phosphorylation of TGF-β or BMP-specific-receptor-regulated Smad proteins (R-Smad). R-Smads then associate with the common mediator (co)-Smad (Smad4) and translocate to the nucleus to regulate the transcription of specific target genes in association with other partner proteins. R-Smads are divided in two groups. The first group consists of Smad1/5/8 and are preferentially activated by BMP type I receptors that include ALK1, 2, 3, and 6. The second group contains Smad2 and 3 and is activated by TGF-β type I receptor ALK5 (Feng and Derynck, 2005; Massagué and Gomis, 2006). TGF-β and BMPs can also activate Mitogen Activated Protein (MAP)-Kinase signaling pathways, Rho-like GTPase and PI3K/Akt cascades independently of Smad signaling pathways (Moustakas and Heldin, 2005).

Genetic studies in mice and humans have clearly demonstrated the importance of TGF-β/BMP signaling pathways in vascular morphogenesis and angiogenesis. Information gathered the various loss-of-function mouse models of TGF-β signaling components have recently been reviewed in detail (Jakobsson and van Meeteren, 2013). In all cases, targeted deletions of *tgfb1* (Dickson et al., 1995), of genes encoding TGF-β receptors, *acvrl1* (Oh et al., 2000; Urness et al., 2000), *Alk5* (Larsson et al., 2001), *TβrII* (Oshima et al., 1996) or *Eng* (Bourdeau et al., 1999, 2000; Li et al., 1999; Arthur et al., 2000) as well as the downstream target *Smad5* (Chang et al., 1999; Yang et al., 1999) lead to embryonic lethality at mid gestation with severe cardiovascular defects that include impaired angiogenesis and differentiation of mural cells (Table 1). The primary target cells for TGF-β/BMP are endothelial cells since mice deficient in endothelial TβRII (Carvalho et al., 2007), ALK1 (Garrido-Martin et al., 2014; Tual-Chalot et al., 2014), ALK5 (Carvalho et al., 2007) or endoglin (Mahmoud et al., 2010; Garrido-Martin et al., 2014) show various vascular defects ranging from vessel hyper-branching, enlarged blood vessels to AVM formation. The involvement and activity of these TGF-β/BMP signaling components are strictly linked to the development stage (Table 1) (Jakobsson and van Meeteren, 2013).

**Table 1:**
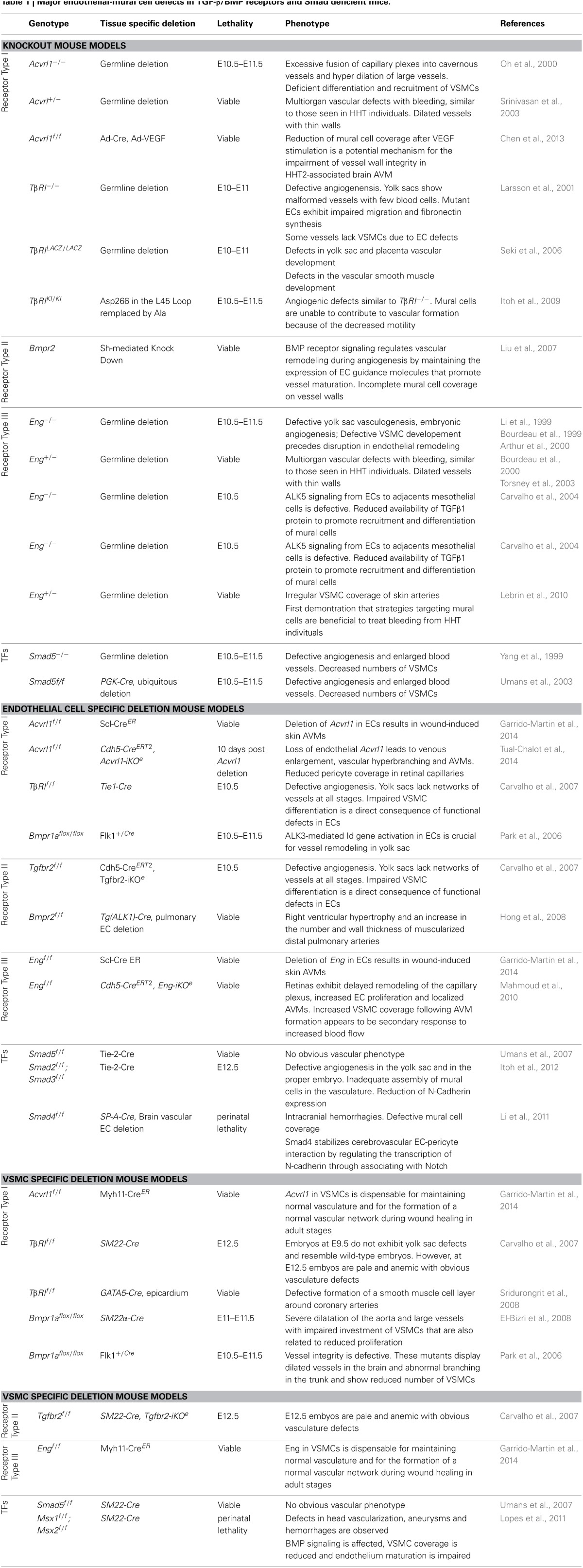
**Major endothelial-mural cell defects in TGF-β/BMP receptors and Smad deficient mice**.

Interestingly, impaired TGF-β/BMP signaling pathways not only affect endothelial cells but they are also important for proper recruitment and differentiation of mural cells (Table 1). Moreover, mural cell specific deletion of TGF-β/BMP components are linked to vascular defects but at later stages of development indicating that TGF-β/BMP signaling pathways regulate vessel remodeling (Table 1) (Carvalho et al., 2007). One important issue over the past decade has been to identify whether the mural cell defects observed in the TGF-β mutants reflected the primary effects of TGF-β signaling in mural cells or occurred secondarily to the impairment of endothelial cell functions (Table 1). TGF-β has been proposed to regulate the activation state of endothelial cells by differentially activating two TGF-β type I receptors, ALK5 and ALK1. ALK5 is broadly expressed in almost all tissues whereas ALK1 is restricted to the endothelium. Upon TGF-β stimulation, ALK5 phosphorylates Smad2/3 leading to inhibition of endothelial cell proliferation and migration, whereas ALK1 phosphorylates Smad1/5 to induce opposite effects (Goumans et al., 2002). The existence of two type I receptors activated by one ligand, raises the question of how their activation is controlled and why these two cascades coincide. Although not experimentally proven, one explanation is that ALK1 signaling first may dominate, leading to the activation phase of angiogenesis triggered by VEGF, whereas ALK5 may induce later vessel stabilization and extracellular matrix production. However, ALK5 kinase activity seems to be required for optimal ALK1 signaling whereas ALK1/Smad1/5 signaling directly antagonizes ALK5/Smad2/3 signaling cascade (Goumans et al., 2003; Itoh et al., 2009). The net effect of TGF-β may therefore depend on the relative levels of ALK1/ALK5 expression and also on the different levels of TGF-β (Goumans et al., 2002). The type III TGF-β co-receptor endoglin is highly expressed on activated endothelial cells. It is required for efficient ALK1 signaling. Interestingly, endothelial cells lacking endoglin do not proliferate due to enhanced ALK5 signaling cascade. Endoglin may therefore regulate fine-tuning between ALK1 and ALK5 activated cascades (Lebrin et al., 2004; Blanco et al., 2005). Both constitutive and conditional deletion of *Acvrl1* or *Eng* in the endothelial cells lead to impaired angiogenesis and the development of vascular malformations indicating that these receptors share functions in signaling (Allinson et al., 2007; Park et al., 2009; Mahmoud et al., 2010; Garrido-Martin et al., 2014; Tual-Chalot et al., 2014). The situation is yet more complex for ALK5 since conflicting data exist on its expression pattern in the endothelium. Using an *acvrl1* (ALK1)-Cre to delete TβRII and ALK5 specifically in the endothelium, Park et al. have suggested that the effects of these receptors on vessel morphogenesis and angiogenesis were not due to their functions in endothelial cells (Park et al., 2008) supporting that ALK5 expression preferentially occurs in mural cells as suggested by Seki et al. (2006). However, floxed *TβRII* and floxed *Alk5* mice crossed with transgenic mice expressing the Cre-recombinase under control of the vascular endothelial specific Tie1 promoter resulted in embryonic lethality at E10.5 because of aberrant angiogenesis as for the conventional *TβRII* and *Alk5* knockout mice (Carvalho et al., 2007). Another Cre-driver where EGFP-Cre was knocked into the *acvrl1* (active at E9.5 in the endothelium) to delete TβRII and Alk5 also led to severe blood vessel anomalies and intracranial hemorrhages (Nguyen et al., 2011). The temporal regulation of the promoters used indicate that ALK5 expression in endothelial cells may be required for angiogenesis only at certain developmental stages and may be dispensable for the maintenance of the mature vasculature. Whilst, this hypothesis awaits further experiments for support, ALK1 is known to trigger Smad1/5 pathway upon BMP9 or BMP10 stimulation to induce blood vessel quiescence (David et al., 2007, 2008; Scharpfenecker et al., 2007; Ricard et al., 2012). *Bmp9*^−/−^ and *BMP10*^−/−^ mice do not display lethal defects in yolk sac development (Chen et al., 2004; Ricard et al., 2012), but they seem to be important in postnatal remodeling of the retina (Ricard et al., 2012).

The primary cause of HHT may thus be considered as dysfunctions of TGF-β and BMP in endothelial cells. However, how defects in the delicate balance between TGF-β/ALK1-endoglin/ALK5 and BMP/ALK1-endoglin signaling lead to disease pathology remains to be clarified. *Eng*^+/−^ or *Acvrl1*^+/−^ mutant mice are useful models that develop age-dependent vascular lesions similar to those seen in HHT individuals (Bourdeau et al., 2001; Srinivasan et al., 2003; Torsney et al., 2003). Several different studies have characterized these models for their responses to TGF-β. They found that both TGF-β/ALK1 and TGF-β/ALK5 signaling cascades were impaired and ALK5 expression levels were reduced (Lebrin et al., 2004; Xu et al., 2004). These results were further confirmed in blood outgrowth endothelial cells isolated from HHT individuals (Fernandez-L et al., 2005). The mechanisms leading to decrease ALK5 expression remain to be clarified although it is suspected to be a consequence of a transcriptional modulation by ALK1 signaling (Fernandez-L et al., 2005). ALK5 promotes vessel maturation favoring cell growth arrest, differentiation and extracellular matrix production suggesting that reduced expression of endoglin or ALK1 may result in the inability of blood vessels to mature properly. Although not experimentally proven, it is likely that similar mechanisms might be operative in the context of BMP9. Indeed, some Smad4 mutations, the common mediator of all R-Smad-dependent TGF-β/BMP family signaling can cause a syndrome that includes both juvenile polyposis and HHT (Gallione et al., 2004, 2006). Li et al. have recently reported that endothelial-specific deletion of *Smad4* resulted in blood brain barrier breakdown with severe hemorrhages. These mutants exhibited vascular dilation and reduced pericyte coverage (Li et al., 2011). Interestingly, they found that Smad4 and Notch signaling act in concert to regulate the expression of N-Cadherin, a cell-adhesion molecule that mediates heterotopic cell contacts between endothelial cells and pericytes (Gerhardt et al., 2000; Gerhardt and Betsholtz, 2003). Deletion of *Smad2* and *Smad3* specifically in the endothelial cells led to embryonic lethality at E12.5 due to inadequate assembly of mural cells to the vasculature. This phenotype was also linked to reduced expression of N-Cadherin as well as Sphingosine-1-Phosphate Receptor 1 (S1PR1) (Itoh et al., 2012). In summary, HHT may result from a general defect in TGF-β/BMP in endothelial cells affecting mural cell attachment and vessel stabilization. The endothelium will be more prone to respond to angiogenic stimulus leading to excessive sprouting of vessels with attendant formation of AVMs (Park et al., 2009; Lebrin et al., 2010; Mahmoud et al., 2010; Chen et al., 2013; Choi et al., 2013).

## Impaired TGF-β activation affects pericyte differentiation

TGF-β isoforms (TGF-β1, TGF-β2, and TGF-β3) are secreted in latent forms that need to be activated before they can bind to their receptors. Both pericytes and endothelial cells express TGF-β, although its activation requires a close physical association with the endothelium through gap junctions (Sato and Rifkin, 1989). Gap junctions are aggregates of intercellular channels that allow the diffusion of second messengers and metabolites to the cytoplasm of adjoining cells. Genetic studies in mice have revealed critical roles for Connexin (Cx) Cx43 and Cx45 in the regulation of endothelial-mural cell differentiation by promoting TGF-β activation (Krüger et al., 2000; Hirschi et al., 2003; Fang et al., 2013). Other gene deletions/mutations may also result in vascular phenotypes because of interactions with TGF-β activation. They include Tissue factor, a pro-coagulant receptor (Carmeliet et al., 1996) and integrins such as α_*v*_β_8_(Bader et al., 1998; Zhu et al., 2002; Cambier et al., 2005). It is not yet clear how integrins regulate TGF-β activation but it might require Matrix Metalloproteinases (MMPs) (Mu et al., 2002; Cambier et al., 2005) and/or cell constriction mediated tensile force across latent-TGF-β (Shi et al., 2011). More recently, it has been suggested that α_*v*_β_8_ may induce a gradient of activated TGF-β in the CNS, which in turn suppresses sprouting angiogenesis thereby stabilizing blood vessels (Arnold et al., 2014).

Importantly, the absence of endoglin in endothelial cells results in reduced phosphorylation of Smad2 in the adjacent mural cell layer as a consequence of defective TGF-β activation (Carvalho et al., 2004) whereas reduced endothelial secretion and plasma levels of TGF-β have been reported in HHT1 individuals (Letarte et al., 2005). Local activation of TGF-β may thus be compromised in HHT individuals due to defective interaction between pericytes and endothelial cells affecting mural cell differentiation and vessel stabilization that are typical clinical symptoms of HHT (Torsney et al., 2003; Lebrin et al., 2010; Chen et al., 2013; Tual-Chalot et al., 2014).

Pericytes only express ALK5 (Van Geest et al., 2010). Upon activation by TGF-β, ALK5 leads to the phosphorylation of Smad2/3 that induces production of contractile proteins, cell quiescence and differentiation (Owens, 1998; Van Geest et al., 2010). TGF-β through Smad3 and p38MAPK increases the expression of α-SMA and smooth muscle myosin (Seay et al., 2005). Specific deletion of *TβrII* and *Alk5* in mural cells leads to embryonic lethality between E12.5 and E16.5, which is slightly later than conventional knockout mice (Carvalho et al., 2007). Together, these data suggest that TGF-β is required during the later phase of angiogenesis to induce pericyte differentiation and vessel maturation following recruitment of the mural cells by PDGF-B.

BMPs also play a role in VSMC differentiation and functions at least through the regulation of *Msx* genes (Yu et al., 2005, 2008; Lopes et al., 2011). *Alk3* conditional knockout mice where the receptor is deleted in flk1 precursors displayed multiple abnormalities in vascular development including vessel remodeling and maturation (Park et al., 2006). Mutations in *BMPR2* result in pulmonary Hypertension (PAH), a vascular disorder characterized by uncontrolled remodeling of the pulmonary arteries due to increased proliferation of VSMCs (Beppu et al., 2004). The link between deregulated BMP signaling, pericyte-endothelial cell communication and HHT disease progression remains to be determined but an important clue may come from the fact that HHT individuals who have *ACVRL1* mutations are predisposed to the development of PAH (Girerd et al., 2010; Gore et al., 2014).

In the light of these results, the unique HHT genetic models recently generated may potentially facilitate future studies to better understand the molecular mechanisms regulating pericyte endothelial cell communication and importantly, how defective communication between these two cell types is involved in HHT pathogenesis (Table 1).

## Targeting pericytes to stimulate vessel maturation in HHT

Could the signaling pathways involved in endothelial-mural signaling crosstalk provide new drug targets in HHT? Recently, we have revealed a novel mechanism of action of thalidomide, namely stimulation of vessel maturation and have reported that oral administration of thalidomide reduced both the frequency and duration of nosebleeds with significant decreases of blood transfusion requirement and improvement of quality of life (Lebrin et al., 2010). Few other cases have been reported so far, but the published literature is concordant regarding the potential benefit of thalidomide in HHT individuals (Table 2). All subjects treated with thalidomide had severe and recurrent epistaxis and they were refractory individuals to standard medical and local surgical treatments. Overall, thalidomide was administrated orally and the doses given were comparable to that prescribed in the 1960s to treat nausea in pregnancy, ranging from 50 to 300 mg of thalidomide daily. In most cases, only minor side effects have been reported and include mild constipation, loss of libido, drowsiness and lethargy. However, three individuals stopped treatment due to peripheral neuropathies in two individuals and deep vein thrombosis in one subject (Table 2). Therefore, thalidomide appears to be a potential candidate for the treatment of severe bleeding in HHT individuals unresponsive to conventional therapies. However, these studies have not yet been supported by data from randomized controlled trials and future research should be directed toward identifying the minimum dose of thalidomide effective to prevent bleeding from HHT vascular anomalies without inducing side effects.

**Table 2:** **Thalidomide prevents bleeding from HHT individuals**.

**No. of patients**	**Follow-up period (months)**	**Sex/Age (years)**	**Thalidomide dose (mg/daily)**	**Haemoglobin level (mmol/ml)**		**Number of bleedings (per week)**		**Duration of bleeding**		**Number of transfusions**		**Side effects**	**References**
				**Before**	**After**	**Before**	**After**	**Before**	**After**	**Before**	**After (during follow-up)**		
1	13	M/77	150–250	NA	NA	21–28	3–4	45–90	1–2	NA	NA	Mild peripheral neuropathy I the finger and toes	Kurstin, 2002
												Mild sedative effects	
8	6	NR/NR (57–69)	100–300	4.8–7.7	6.2–12.2	NA	NA	NA	NA	2–10 blood units/months	0–5 blood units/months	Minor side effects (constipation and vertigo) expect for one patient who stopped after 4 months because of poor responses and side effects	Buscarini et al., 2009
4	6	NR/NR	50–200	NA	NA	NA	decrease 54–89%	NA	NA	2–10 blood units/year	19–57% decrese	No serious complications (drowsiness)	Gossage et al., 2009
7	6–60	6 M/1 F/60 (43–75)	100	4.3–7.2	6.2–8.7	18–59	1–35	10–90	<5–20	0–8	0–NA	Mild consipation, loss of libido, drowsiness, lethargy. One individual stopped treatment after 19 months because of peripheral neuropathy	Lebrin et al., 2010
1	1	F/59	100	NA	NA	NA	NA	NA	NA	NA	NA	Deep vein trombosis	Penaloza et al., 2011
1	16	F/77	100	5.8	13.0	NA	NA	NA	NA	24–36 blood units/year	<1 blood units/year	NA	Alam et al., 2011

*Case reports from the literature*.

Thalidomide was first introduced as a sedative used to prevent nausea during pregnancy in the late 1950s. In 1961, it was withdrawn from the market due to teratogenicity and neuropathy (Speirs, 1962). The use of thalidomide resulted in one of the biggest tragedy in the history of drug development. As a result of using thalidomide, it caused an estimated 10,000 children in 46 countries to be born with birth defects, marked by limb malformations and congenital defects affecting ears, eyes, heart and kidney. These defects occurred when drug exposure took place within a short, time-sensitive window between day 20 and day 36 of gestation. Just one 100-mg tablet of thalidomide was enough to cause limb defects (D'Amato et al., 1994; Therapontos et al., 2009). This drug was abandoned but has recently undergone a renaissance. Emerging insight into thalidomide's anti-inflammatory, immunomodulatory and anti-angiogenic activity led to clinical trials in AIDS-related aphthous ulceration, Behcet's syndrome, Crohn's disease cutaneous lupus and various malignancies (Shortt et al., 2013). In 1999, effectiveness against multiple myeloma (MM) was reported (Singhal et al., 1999). In respect to Erythema Nodosum Leprosum (ENL) and MM, the US FDA approved thalidomide for use under strict guidelines and carefully controlled inclusion criteria in 1998 and 2006, respectively. Decade of investigation have identified a multitude of biological effects that are regulated by thalidomide. In addition to suppression of Tumor Necrosis Factor-α (TNF-α), thalidomide affects the generation and elaboration of a cascade of pro-inflammatory cytokines that activate cytotoxic T-cells even in absence of co-stimulatory signals. Furthermore, VEGF and basic Fibroblast Growth Factor (bFGF) secretion and cellular response are suppressed by thalidomide, thus antagonizing angiogenesis and altering the bone marrow stromal microenvironment in hematologic malignancies (Melchert and List, 2007; Shortt et al., 2013). More recently, preclinical studies have identified E3 ligase protein cereblon (CRBN) as a direct molecular target for the teratogenicity of thalidomide (Ito et al., 2010). CRBN is also required for the anti-myeloma activity of thalidomide (Zhu et al., 2013).

Interestingly, we have reported that the anti-hemorrhagic property of thalidomide is not the result of direct inhibition of endothelial cell proliferation and migration but is rather due to increased mural cell coverage of the vasculature. Thalidomide increased the number of pericytes and their recruitment to blood vessels, enhancing the apposition between the inner endothelial and supportive pericyte layers and resulting in vessel stabilization in *Eng*^+/−^ mutant mice, a well-characterized model of HHT. Moreover, high doses of thalidomide (150 mg/Kg body weight) stimulated the number of pericytes that expressed α-SMA, an established marker of the pericyte contractile phenotype. At the molecular level, thalidomide-treated mouse retinas unexpectedly showed only marginally reduced *VEGF* mRNA levels compared to untreated controls. However, we observed a marked and rapid increase of *PDGF*-β mRNA levels in endothelial cells in response to thalidomide. PDGF-B is a key molecule in pericyte chemotaxis that promotes endothelial-pericyte cell-cell contact. The observation that the anti-angiogenic effects of thalidomide were prevented by concurrent administration of imatinib, a kinase inhibitor that blocks PDGFR-β but not VEGF signaling suggests a functional role for PDGF-B in this thalidomide-stimulated reduction in angiogenesis. Moreover, we took advantage of the *Pdgf*^*ret/ret*^ mouse model in which PDGF-B is secreted but is not retained by the extracellular matrix and so does not form the gradient required to stimulate tight adhesion of pericytes to the abluminal surface of microvessels and showed that thalidomide did not rescue the pericyte recruitment defect in post-natal *Pdgf*^*ret/ret*^ mice. Finally, we revealed that thalidomide might target mural cells directly to stimulate their proliferation and ability to form protrusions independently of effects on PDGF-B signaling. The exact mechanisms underlying this effect need further investigation. Our data provide to our knowledge, the first evidence that a therapy targeting pericytes to stimulate vessel maturation can have beneficial effects on bleeding from vascular malformations (Lebrin et al., 2010).

## Perspectives

**Figure 2:**
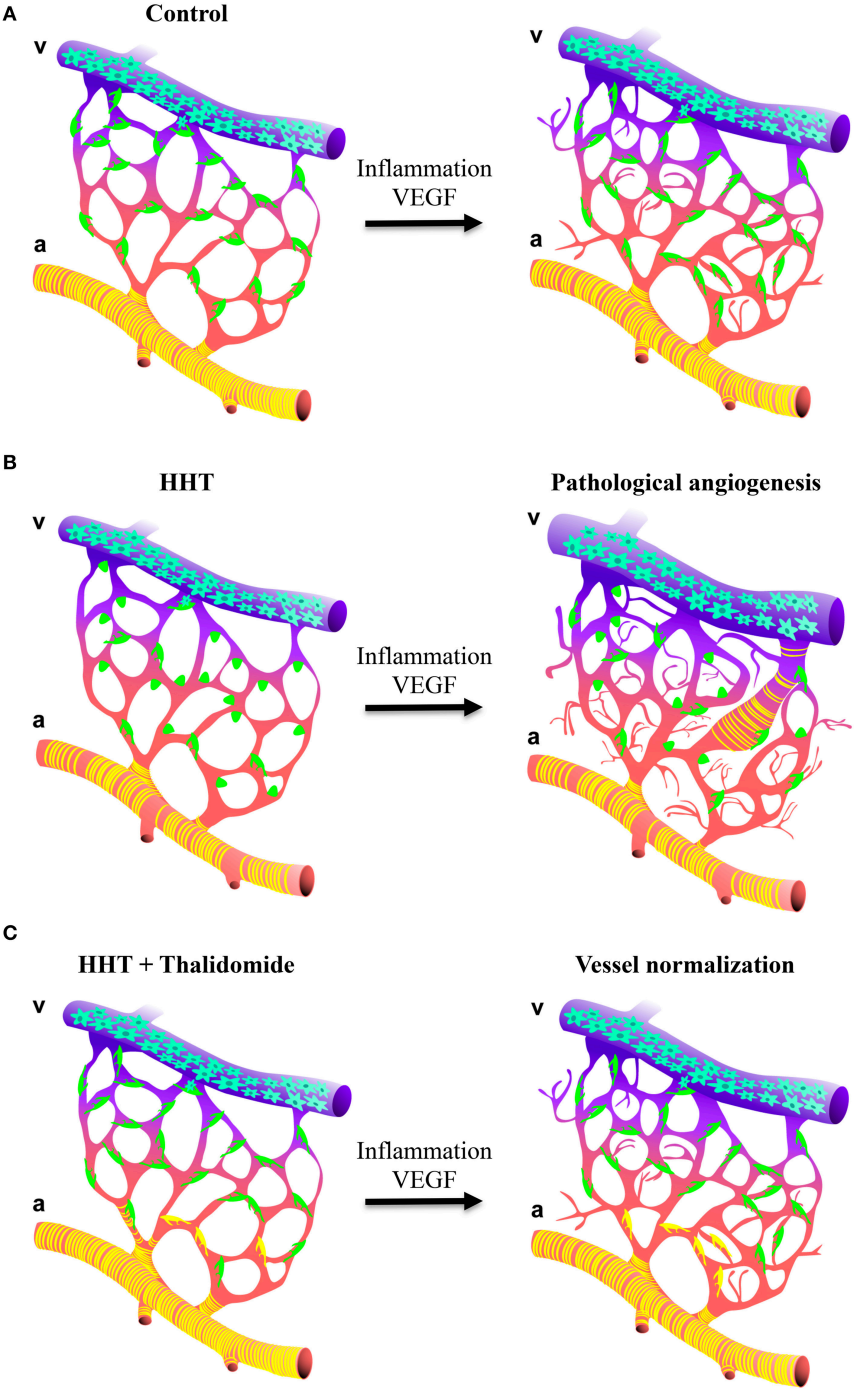
**Schematic illustration of how thalidomide prevents excessive angiogenesis in HHT. (A,B, left panels)** Impaired TGF-β/BMP in endothelial cells affects mural cell attachment and vessel stabilization. Blood capillaries show endothelial hyperplasia and irregular capillary diameter. **(A,B, right panels)** As consequence, blood vessels from HHT are more sensitive to angiogenic stimulus i.e., inflammation or VEGF and develop a broad spectrum of vascular abnormalities ranging from excessive angiogenesis, chaotic vascular organization and the formation of AVMs. **(C)** Thalidomide increases mural cell coverage to sustain the quiescence of the vasculature. As consequence, the blood vessels are less sensitive to angiogenic stimulus preventing excessive angiogenesis and the formation of vessel anomalies.

Our understanding of why the disease gene mutations lead to the vascular pathology is finally advancing and suggests that HHT mutations may be deleterious predominantly in endothelial cells with specific effects on the communication between pericytes and endothelial cells leading to vessel instability. Whilst mural cells will be recruited to the vessels, impaired TGF-β/BMP signaling in endothelial cells will result in poor attachment of the mural cells to the endothelium leading to defective TGF-β activation and subsequently poor mural cell differentiation. These vascular abnormalities will coincide with abnormally variable capillary diameters and vessels that are more prone to respond to angiogenic stimuli. This model implies that activation of the quiescent endothelium, for example by inflammation and/or angiogenesis i.e., wounding or VEGF stimulus, will induce excessive vessel sprouting and the development of a broad spectrum of vascular abnormalities such as AVMs the pathological hallmark of HHT (Figure 2; Park et al., 2009; Lebrin et al., 2010; Mahmoud et al., 2010; Chen et al., 2013; Choi et al., 2013). In such context, defective endothelial cell-pericyte communication may promote AVM formation by different mechanisms: abnormal vascular remodeling and dilation following inflammation or trauma may fail to resolve; increased number of regressing vessels would remove the capillary bed that separates arteries and veins; or gradual dilation of an anastomosis may occur as a result of loss of mural cells and/or loss of vessel tone leading to capillary regression due to the lack of blood flow.

These exciting insights into the complex molecular signaling cascades governing endothelial-pericyte interactions in the context of HHT have raised several important questions. For instance, what are the exact mechanisms underlying TGF-β activation or on the other hand, are known mediators of TGF-β activation deregulated in HHT models? If differential regulation of multiple signaling pathways such as Notch signaling does occur, how these signaling pathways are affected in HHT? Are they defective only in specific vascular beds? What are the contributions of the altered BM protein composition to the HHT phenotype? It is also likely that defective mural cell attachment and maturation in HHT will have consequences not only during angiogenesis and vessel remodeling but also on the ability of the capillaries to regulate blood flow and vessel permeability to solutes and cells, important functions that require pericytes (Winkler et al., 2011). However, data to support this hypothesis are still lacking.

Our findings indicate that strategies targeting pericyte-endothelial cell communication to stimulate vessel maturation can have beneficial effects on bleeding by normalizing the vessel anomalies (Figure 2; Lebrin et al., 2010). Thalidomide reduces nosebleeds in HHT individuals in part by enhancing PDGF-B expression that leads to the recruitment of mural cells and in part through unknown mechanisms. Both inflammation (Mahmoud et al., 2010) and mononuclear cells (van Laake et al., 2006) have been ascribed to account for the development of vascular malformations in HHT. It would therefore be important to determine whether the anti-inflammatory and immunomodulatory properties of thalidomide may also contribute to the beneficial effects of this drug. Thalidomide treatment is not without risk since it has poor specificity, affecting a range of physiological processes and has side effects (Shortt et al., 2013). It might therefore not ultimately be the drug of choice for the treatment of HHT. However, due to the encouraging activity of thalidomide in MM, many analogs have been developed to be more potent and specific than thalidomide. A class of agents, termed the immunomodulatory drugs (IMiDs) represents promising compounds for the treatment of cancers. Some are under clinical investigation and CC-5013 (lenalinomide) and CC-4047 (Pomalinomide, Actimid) have obtained FDA-approvals for 5q-myelodysplasia and for MM (Shortt et al., 2013). Understanding the mechanisms of action by which thalidomide stimulates vessel maturation will help to design new drugs targeting pericytes that have fewer side effects, leading to new therapeutic options for HHT individuals. More generally, strategies targeting pericytes to stimulate vessel maturation may open new avenues to treat pericyte-associated diseases such as diabetes, cancers and neurodegenerative disorders.

### Conflict of interest statement

The authors declare that the research was conducted in the absence of any commercial or financial relationships that could be construed as a potential conflict of interest.
